# Isolated stridor without any other sleeping breathing disorder diagnosed using drug-induced sleep endoscopy in a patient with multiple system atrophy: A case report

**DOI:** 10.1097/MD.0000000000019745

**Published:** 2020-04-17

**Authors:** Sung Jae Heo, Jung Soo Kim, Byung Joo Lee, Donghwi Park

**Affiliations:** a Department of Otorhinolaryngology-Head and Neck Surgery, School of Medicine, Kyungpook National University, Kyungpook National University Chilgok Hospital; b Department of Otorhinolaryngology-Head and Neck Surgery, School of Medicine, Kyungpook National University, Kyungpook National University Hospital; c Department of Rehabilitation Medicine, Daegu Fatima Hospital, Daegu, South Korea; d Department of Physical Medicine and Rehabilitation, Ulsan University Hospital, University of Ulsan College of Medicine, Ulsan, Republic of Korea.

**Keywords:** endoscopes, multiple system atrophy, sleep apnea syndrome, stridor

## Abstract

**Rationale:**

Multiple system atrophy (MSA) is a rare neurodegenerative disease characterized by Parkinsonism and autonomic dysfunction or cerebellar ataxia. MSA can be accompanied by stridor caused by laryngeal stenosis secondary to vocal cord dysfunction.

**Patient concern:**

A 60-year-old woman with MSA, complaining of difficulty in breathing during sleep. Her bed partner reported witnessing grunting-like sounds during sleep.

**Diagnosis:**

Isolated stridor without any other sleeping breathing disorder diagnosed using drug-induced sleep endoscopy (DISE) in a patient with MSA.

**Interventions:**

On polysomnography, there was no obstructive sleep apnea. Using DISE, abnormally adducted vocal cords during inspiratory respiration were identified, leading to a diagnosis of stridor. We prescribed positive airway pressure to resolve the stridor.

**Outcome:**

Our patient was also prescribed continuous positive airway pressure for the treatment of nocturnal stridor, and it is improved.

**Lessons:**

In summary, when MSA patients present with nocturnal stridor, it is important to evaluate the exact diagnosis and cause of stridor in patients by confirming the movement of vocal cords using DISE, as well as polysomnography.

## 1 Introduction

Multiple system atrophy (MSA) is a rare neurodegenerative disease characterized by parkinsonism and autonomic dysfunction or cerebellar ataxia.^[1]^ MSA can be accompanied by stridor, which is known to be caused by laryngeal stenosis secondary to vocal cord dysfunction.^[2]^ In patients with MSA, stridor is associated with respiratory failure and sudden death during sleep.^[2]^ Therefore, it is important to identify it early and find appropriate treatment. Stridor is an easily recognizable harsh and strained high-pitched sound, which is usually inspiratory, but can be expiratory or biphasic. Therefore, audio recording during polysomnography (PSG) is typically used for a diagnosis of stridor.^[3]^ However, audio analysis has the disadvantage that it cannot accurately diagnose the cause of stridor. Drug-induced sleep endoscopy (DISE), which has become an invaluable tool in the evaluation of patients with sleep-disordered breathing (SDB), involves the use of a flexible nasopharyngoscope to evaluate the upper airway during the drug-induced sleep state.^[4,5]^ In contrast to PSG; therefore, DISE may provide a more direct assessment of the behavior of patients’ upper airway anatomy during sleep, which can provide clues to the cause of stridor. However, most studies examining the utilization of DISE in the management of patients with SDB have been performed only in patients with obstructive sleep apnea (OSA). Thus, until to date, few studies have reported the utility of DISE in the diagnosis of patients with stridor. In addition, most cases of stridor in MSA patients tend to be accompanied by other SDB, such as OSA or central sleep apnea; therefore, isolated stridor is rare in MSA patients.^[3]^ Therefore, we report a case of isolated stridor without any other sleeping breathing disorder diagnosed using DISE in a patient with MSA.

## 2 Report of case

**Figure 1:**
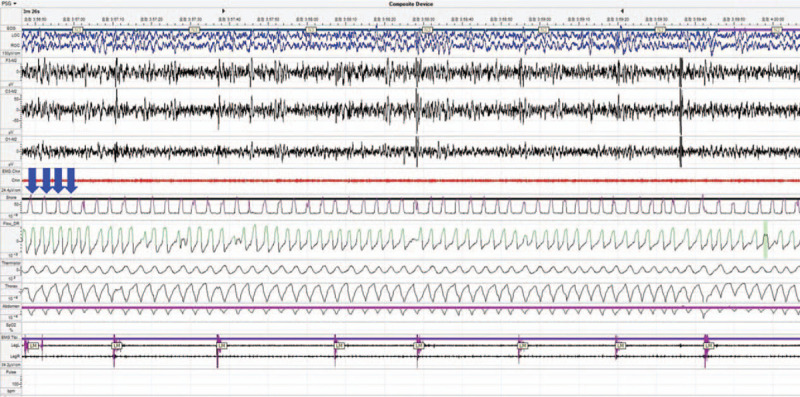
Night polysomnography shows a repeated high-pitched inspiratory sound (arrow).

A 60-year-old woman complaining of difficulty in breathing during sleep visited our department. Patient has provided informed consent for publication of the case. Her bed partner reported witnessing grunting-like breathing sounds and that she woke up frequently during sleep. She has been diagnosed with MSA and diabetes and was under treatment at the neurology department. She underwent type I PSG to diagnose OSA. The PSG found an apnea-hypopnea index of 0.6/h (apnea index and hypopnea index were 0/h and 0.6/h, respectively). As a result, her symptom was not related to OSA, in contrast to our expectations. During PSG examination, she made abnormal inspiratory breathing sounds like stridor (Fig. 1). Because we could not confirm the identity or cause of the sound with PSG, we performed DISE to evaluate the obstruction pattern of the upper airway.

**Figure 2:**
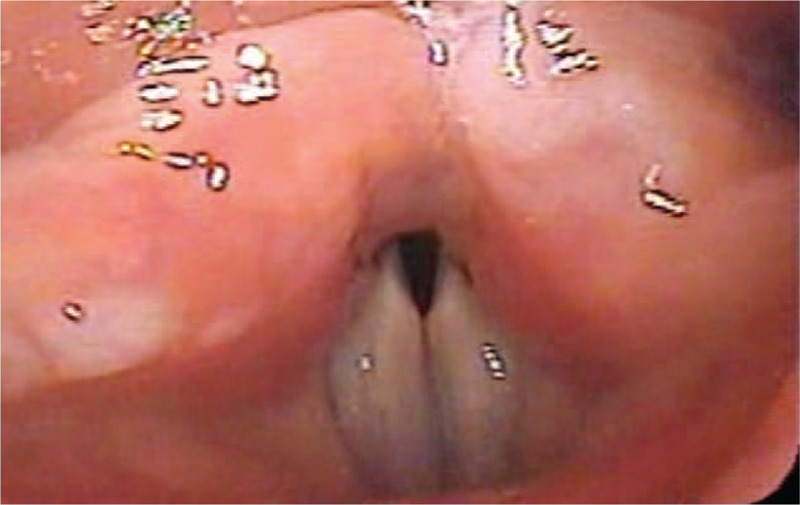
Abnormally adducted vocal cords during DISE were observed. DISE = drug-induced sleep endoscopy.

DISE was performed in the outpatient clinic by an Otolaryngologist specialist. The nasal mucosa was topically anesthetized and shrunk using gauze soaked with 4% lidocaine and 0.1% epinephrine. After sufficient nasal anesthesia, the patient was placed in the supine position and peripheral oxygen saturation was monitored using a pulse oximeter. The bispectral index was used to monitor the depth of sleep. Midazolam at a dose of 0.05 mg/kg was administered intravenously to induce sleep and added up to 9 mg under careful monitoring, if needed. A nasopharyngoscope (VNL1130, KayPENTAX, Montvale, NJ) was inserted through the wider nasal cavity at the onset of SDB.^[5]^ The patient fell asleep with midazolam 3.5 mg and began to make an inspiratory harsh stridor sound. Common obstruction sites for SDB such as the velum, lateral pharyngeal wall, tongue base, and epiglottis were patent. However, the vocal cord was adducted during inspiratory respiration (Fig. 2) and produced stridor. She did not have any dysfunction of vocal cord movement or respiratory problems while awake; it happened only during sleep. We prescribed continuous positive airway pressure (CPAP) to resolve the stridor. The titrated pressure of CPAP was 6.0 cm H_2_O and median air leakage was 8.4 L/min. No apnea and hypopnea were observed while wearing a CPAP. During a year of follow-up period, she was well treated with CPAP without complications or discomfort.

## 3 Discussion

Stridor in MSA patients may also be confused with heavy snoring by automatic sleep devices; hence, sleep studies should include an audio recording of raw sounds that could be heard by someone who has been trained to recognize this typical, inspiratory harsh-pitched sound, mostly present in N3 sleep.^[3]^ Thus, the isolated usage of audio in the diagnosis of stridor may be difficult. The mechanism of stridor may not be fully understood by using audio analysis alone during PSG.

On laryngoscopy, patients with MSA typically showed limited laryngeal movements, even during an alert state.^[6]^ However, the movement of the vocal cords may be normal when patients have only nocturnal stridor, as in this case. Nonetheless, the use of DISE may offer clues to the underlying mechanism of nocturnal stridor, even if there are no abnormalities during the alert state. In patients with MSA, stridor during sleep occurs mainly due to hyperactivity of the vocal cord adductor muscles and low activity of the vocal cord abductor, resulting in an increased resistance of the upper larynx due to impairment of cerebrospinal function controlling the activity of the laryngeal nerve.^[2]^ In addition, it is known that the mechanism of stridor generation only during sleep is the decrease of nervous stimulation of the upper larynx dilator muscles during sleep.^[2]^

MSA patients with stridor have bad quality of life.^[7]^ Stridor affects survival of MSA patient due to respiratory failure and sudden death.^[2]^ The earliest treatment for stridor was surgical (including tracheostomy, tracheotomy, and cordotomy), until previous studies reported that the application of CPAP improved nocturnal stridor.^[3,8,9]^ Currently, the recommended treatment of stridor is nasal CPAP and has been reported to improve the quality of life in MSA patients with stridor.^[3]^ Our patient was also prescribed CPAP for the treatment of nocturnal stridor. In summary, when MSA patients present with nocturnal stridor, it is important to evaluate the exact diagnosis and cause of stridor in patients by confirming the movement of vocal cord using DISE, as well as PSG.

## Author contributions

**Conceptualization:** Jung Soo Kim.

**Writing – original draft:** Sung Jae Heo, Donghwi Park.

**Writing – review & editing:** Sung Jae Heo, Byung Joo Lee, Donghwi Park.
